# On the limits of the intervention on complex systems guided by functional networks

**DOI:** 10.1038/s41598-025-08933-z

**Published:** 2025-07-01

**Authors:** Massimiliano Zanin

**Affiliations:** Instituto de Física Interdisciplinar y Sistemas Complejos (CSIC-UIB), Campus UIB, 07122 Palma, Spain

**Keywords:** Complex networks, Statistics

## Abstract

Complex networks, and functional networks in particular, have become a standard tool to understand the structure and dynamics of real-world complex systems. One usually hidden assumption is that the structure of the reconstructed functional networks encodes useful information to guide interventions on the physical layer, when the latter is not known. We here test this assumption using a minimal model, simulating a propagation process in a physical network, and guiding interventions using node properties observed in the corresponding functional representation. We show how this approach becomes less optimal the more complex the topology is; up to becoming marginally better than choosing nodes at random in the real case of the European air transport network.

## Introduction

Complex networks theory^1–3^ has established itself as a foundational tool for the study of complex systems, both natural and manmade. This is explained, on the one hand, by its capacity to describe in a synthetic manner the patterns of interconnections between the composing elements; but also, on the other hand, to support the understanding of the processes developing on top of them. This last element is critical in many real-world scenarios, as the function of a system is usually connected to the movement of some properties, objects or signals within it^4^. To illustrate, social networks support the spreading of information^5^, but also of diseases^6^, among the participating individuals; financial and trade markets, the movement of money and commodities^7^; transportation networks, the mobility of people and products^8^; and so forth. These connections are not always known, and the researcher has to resort to so-called “functional networks”, i.e. networks in which links are reconstructed from the dynamics of the individual elements, under the assumption that the dynamics of two of them must share some common characteristics whenever the corresponding elements are connected. The functional network approach has allowed important advancements in the study of e.g. the human brain^9^, transportation systems^10^, climate^11,12^, or financial markets^13,14^. At the same time, it not free from limitations and challenges^15^. Many methodological choices, including the way nodes and links are defined, can substantially change the obtained results^16–18^; noise can mask true relationships^19,20^, also leading to a reproducibility problem^21^; and spurious causal links may appear due to confounding effects^22^.

The study of a complex system using the functional network approach must rely on an essential assumption: the reconstructed network must correctly represent the underlying connectivity, and therefore, it can be used to guide interventions in the system. To illustrate, let us suppose the case of a financial market; the reconstruction of the corresponding functional network may be aimed at identifying the weak (or strong) elements in the system, i.e. those that have to be tackled to improve its robustness (or to reduce their power)^23–25^. Yet, this requires that the weakest (or strongest) nodes in the functional representation correspond to the actual weakest (or strongest) elements in the system; as, otherwise, the intervention would be useless, or inefficient at best. Note that this does not only require that the functional network is correctly reconstructed^26,27^; but that it additionally encodes useful information.

This contribution then tackles the question: how representative functional networks are of the underlying dynamics? And, more specifically, to what degree can the former ones drive interventions in the system? Note that the answer is inherently system- and problem-dependent. To address this, we start by proposing a minimal model of the propagation of the item of interest (e.g. information, passengers, etc.) on top of a physical network of known characteristics; next, a functional network is reconstructed from the observed dynamics using the well-known Granger Causality test^28^; and lastly, we analyse whether topological features of nodes in the functional networks can guide an intervention on the physical one. Results that are obtained are highly topology-dependent, but a general conclusion can be drawn: the more complex is the underlying physical network, the less the properties of the functional one are reliable indicators. We illustrate these conclusions with an analysis of delay propagations in the European air transport network, showing that intervening nodes at random is only marginally worse than using properties like the degree or the betweenness of nodes in the functional network.

## The propagation model

### The physical layer

The model here considered is described by a typical network configuration, in which a set of *N* nodes represents the units of interest of the system. These are pairwise connected by links, which, from a physical perspective, represent connections able to support a propagation. To illustrate, in the case of a transportation system, nodes may represent stations or airports. Connections, on the other hand, represent the presence of vehicles connecting them, and hence the capability of transporting passengers, or of propagating delays. As customary in complex network theory, the physical network is defined by an adjacency matrix ${\mathcal {A}}$, of size $N \times N$, whose element $a_{i, j}$ is equal to one when a connection between nodes *i* and *j* exists, and to zero otherwise. Two additional elements are needed to fully describe the physical part. Firstly, each link is described by a quantity $w_{i,j} \in {\mathcal {N}}$ representing the intensity of the propagation process between nodes *i* and *j* - e.g. the number of vehicles connecting those two stations. Secondly, a value $x_i(t)$ is associated to each node *i*, describing the amount of the propagated property that is observed at that node at time step *i*. For the sake of simplicity, $x_i(t)$ is defined between zero and one; in other words, it can be interpreted as the normalised quantity of passengers or information (or of any other element of interest) there present through time.

Given these ingredients, a simple propagation process is modelled. At a time step *t* and for each pairs of nodes *i* and *j*, we suppose that each vehicle traveling between them has a probability of carrying out the propagation equal to $x_i(t-1)$; thus, on average, the expected number of propagating vehicles will be $x_i(t-1) w_{i,j}$. This alters the status of the receiving node, whose $x_j(t)$ is updated according to the fraction of propagating vehicles by it received. The full dynamics is thus given by a stochastic model defined by the update rule:

1 $$\begin{aligned} x_j(t) = \frac{ \sum _i B( w_{i,j}, x_i(t-1) ) a_{i,j} }{ \sum _i w_{i,j} a_{i,j} }, \end{aligned}$$

where *B* is a binomial distribution. For the sake of simplicity, or in the limit of a large number of vehicles, this can be approximated as:

2 $$\begin{aligned} x_j(t) = \frac{ \sum _i x_i(t-1) w_{i,j} a_{i,j} }{ \sum _i w_{i,j} a_{i,j} }. \end{aligned}$$

Note that all results here presented have been computed according to the stochastic model of Eq. (1), with Eq. (2) being only reported for the sake of clarity.

A simple example can help illustrate this model. Suppose a minimal system composed of two nodes connected in a directional line, i.e. $${\mathcal {A}} = \left[ {\begin{smallmatrix} 0 & 1 \\ 0 & 0 \end{smallmatrix}} \right]$$, with the number of vehicles between nodes 1 and 2 set to $w_{1, 2} = 100$. Trivially, if the initial status of both nodes is $x = 0$ at $t = 1$, nothing can be propagated. On the other hand, suppose that we set $x_1(t = 1) = 0.5$; in other words, the node has a $50\%$ probability of propagating the property of interest to outgoing vehicles. The expected number of affected vehicles in the link $1 \rightarrow 2$ will be 50 out of 100, leading to a new value for node 2 of $x_2(t = 2) = 0.5$; conversely, node 1 will not receive any external contribution, hence $x_1(t = 2) = 0$. In other words, the initial input to node 1 is propagated to the second node, as expected.

### The functional layer

As previously introduced, in many real-world systems, the real propagation process cannot directly be observed; the practitioner has instead to resort to functional representations of the same. To that end, the evolution of the status of each node can be interpreted as a time series; a network is then reconstructed by detecting instances of synchronicity (or more generally, of information flows) between pairs of them. Without loss of generality, we here assume that the functional network is also composed of a set of *N* nodes, and that it is described by a functional adjacency matrix ${\mathcal {A}}^f$, whose element $a_{i,j}^f$ is equal to one whenever a statistically significant propagation is observed between nodes *i* and *j*.

Among the many alternatives available to detect functional connections (a topic that has especially received attention in network neuroscience^9^), we here consider the well-known Granger Causality (GC)^28^. This test is an instance of predictive causality^29^, and therefore it gives information about the directionality of the propagation process; at the same time, it presents the advantage of high simplicity and generality, having been applied to uncountable real-world problems. The interested reader can find information about how such test is calculated in many publications, e.g. Refs.^10,27^ in the context of air transport. From this work’s viewpoint, given two input time series $x_i(t)$ and $x_j(t)$, the Granger test yields a *p*-value representing the statistical significance of the presence of a propagation $i \rightarrow t$. When the test is applied to all pairs of nodes, links that are statistically significant are used to create the functional network ${\mathcal {A}}^f$. Note that, while the GC has here been used due to its conceptual simplicity and prevalence, any other alternative could also be considered, provided it similarly yields a *p*-value for each pair of nodes - as will also be explored below.

**Fig. 1:**
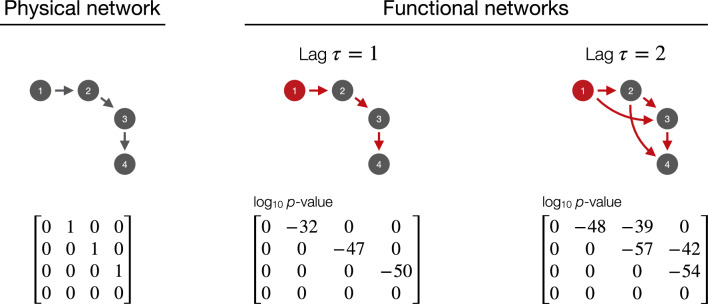
Example of the proposed propagation model. (Left) Physical network composed of four nodes in a chain, including its graphical representation and the corresponding adjacency matrix. (Centre and right) Reconstructed functional networks when the element to be propagated is introduced in the first node (marked in red), for lags $\tau = 1$ (centre) and 2 (right). Red arrows indicate statistically significant propagations according to a GC test; the bottom matrices report all pair-wise *p*-values.

To illustrate the whole reconstruction process, we consider a more complex scenario of a system composed of four nodes connected in a chain - see the left part of Fig. 1, both for a graphical representation of the physical network and the corresponding adjacency matrix. We further suppose that the element of interest is introduced at each time step in the first node with a random amplitude, such that its *x* is drawn from a uniform distribution ${\mathcal {U}}(0, 1)$ - this can represent, for instance, the density of passengers ready to depart from the node. As in the previous case, these elements are expected to flow through the chain. We are nevertheless here adding an additional parameter, specifically the maximum lag $\tau$ in which the GC is evaluated, and representing the maximum number of past time steps used in the calculation of the causality. When $\tau = 1$, only those propagations involving one time step can be detected, and the analysis yields a functional network composed of the chain $1 \rightarrow 2 \rightarrow 3 \rightarrow 4$ - see the central part of Fig. 1. Conversely, for $\tau = 2$, longer propagations are also detected: for instance, node 3 is now influenced by both nodes 1 and 2 - see right part of Fig.1.

This example thus illustrates two points. First, the functional network reconstruction based on GC is able to correctly recover what intuitively may be expected, given that initial physical network. To illustrate, the first node is only propagating, while the last node is always receiving; it thus seems that the properties of the nodes in the functional network are aligned with those in the physical layer. Yet, it is important to note that the resulting functional networks may have topologies that are not the same as the underlying physical one. To illustrate, for a lag of $\tau = 2$, the link density is much higher in the former network. While this is not relevant in the simple problem here considered, this fact will have important consequences in what follows, and will especially impact the effectiveness of interventions in the system.

## Results

### Random physical networks

We start the analysis by considering the simplest structure for a physical network, i.e. a random Erdős-Rényi directed graph of $N = 100$ nodes, with variable link density $d_l$. The propagation strength $w_{i,j}$ is set to random integer numbers drawn from a uniform distribution ${\mathcal {U}}(1, 100)$. We further suppose that the elements to be propagated can appear at random in any one node; in other words, at each time step *t* of the simulation, one node *i* is selected at random, and the corresponding $x_i(t)$ is increased by a random quantity drawn from a uniform distribution ${\mathcal {U}}(0, 0.8)$. Finally, and in order to introduce a dissipation, at each time step all other nodes’ *x*(*t*) are reduced by a $10\%$. The system is let to evolve 1, 000 time steps, in order to collect long-enough time series to be analysed using the GC. In synthesis, this scenario involves the creation and propagation of random elements in a structureless network. In order to extract general conclusions, results below correspond to the average over 200 independent realisations.

**Fig. 2:**
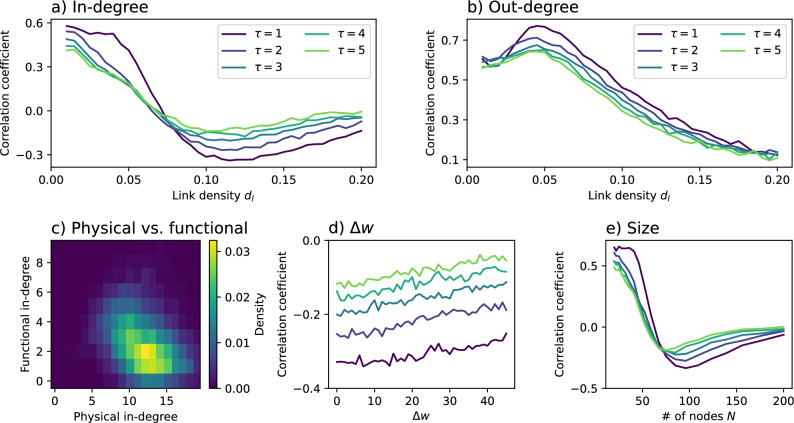
In- and out-degrees in physical random networks. **a**) Evolution of the linear correlation coefficient between the weighted in-degree in the physical networks and the in-degree in the functional networks, as a function of the link density $d_l$, and for different values of $\tau$ (see legend). **b**) Same as the previous panel, but for the out-degree of nodes. **c**) Density of nodes, as a function of their physical and functional in-degrees. **d**) Evolution of the linear correlation coefficient between in-degrees as a function of $\Delta w$, when the number of vehicles in each link is drawn from a distribution ${\mathcal {U}}(1 + \Delta w, 100 - \Delta w)$. **e**) Evolution of the linear correlation coefficient between in-degrees as a function of the number of nodes *N* composing the network. Line colours in panels **d**) and **e**) correspond to different values of $\tau$, see panels **a**) and **b**). Unless otherwise specified, $N=100$, $d_l = 0.12$, and $\tau = 1$. All results correspond to the average over 200 independent realisations.

The first question we pose is whether the most important nodes in the functional representation are the same as the ones in the physical layer. For the sake of simplicity, we start by considering the (possibly) simplest node centrality metrics for the functional networks, i.e. the in- and out-degrees, that is, the number of links arriving to and departing from each node. In the case of the physical network, we consider the weighted version of the same metrics, in order to account for the number of vehicles operating in each connection; the in- and out-degrees for node *i* are then respectively defined as $\sum _j w_{j,i} a_{j,i}$ and $\sum _j w_{i,j} a_{i,j}$. Panels a) and b) of Fig. 2 report the average linear Pearson’s correlation between the degrees of nodes in the two representations, as a function of the link density. Some general trends are easily appreciated, and are in agreement with expectations. Specifically, independently on the lag $\tau$, the correlations tend to zero for dense networks. In other words, in the limit of a fully-connected network, nodes tend to receive from and propagate to all other nodes; as a consequence, the dynamics of nodes becomes almost random, and the GC fails at detecting useful patterns. The correlation is also maximal for low link densities and small values of $\tau$, i.e. very simple and clear structures like the one depicted in Fig. 1. It is nevertheless interesting to observe that a negative correlation emerges for the in-degree for intermediate values of $\tau$; in other words, nodes that are more connected in the physical layer are not necessarily the most receiving ones in the functional layer.

This behaviour is easy to understand by analysing the evolution of the number of inbound links in the functional layer as a function of the same number in the physical layer. As can be seen in panel c) of Fig. 2, when a node is receiving from a large number of sources, its functional in-degree drops to values close to zero. In other words, as the dynamics of the node becomes the sum of many independent inputs, and hence more similar to random, the GC struggles to correctly identify these inputs. Panel d) of the same figure further depicts the evolution of the in-degree correlation coefficient as a function of the variability in the number of vehicles in each link, i.e. when *w* is drawn from a distribution ${\mathcal {U}}(1 + \Delta w, 100 - \Delta w)$; this variability has only a minor effect in the observed negative correlation. On the other hand, the behaviour is strongly modified by the number of nodes *N* in the network, see panel e) of Fig. 2; given a constant link density $d_l$, networks with fewer nodes have also less links, hence a lower probability of displaying the behaviour here discussed.

These results highlight some interesting ideas, applicable when the objective is to understand and manipulate a propagation process only observable through its functional representation, at least in the case of random network structure. While the out-degree in the functional network is a good representation of the number of propagation instances departing from a node, the in-degree counterpart is substantially less reliable. Specifically, when the dynamics of a node is subject to many external inputs, these become difficult to be identified by the GC test, with the result that the node may appear as disconnected. Hence, a node with few functional inbound links may actually be a receiver of many propagation instances. Note that the same does not happen in the case of the out-degree; a node may transmit to many other nodes, but, provided these latter ones are weakly connected, the propagations are still identifiable.

### Interventions in random physical networks

The previous results were based on comparing the centrality of nodes, in the physical and functional representations; yet, caution should be exercised when the objective of the analysis is to guide an intervention on the system. To illustrate, we start from the previous system based on random connectivities and with random appearance of the elements to be propagated; and include a new mechanism according to which, at each time step, the amount of those elements at a given fixed node is reduced by half. This is thus equivalent to an intervention aimed at providing resources to a single node, to minimise its role in the propagation process; the problem then becomes the identification of the best node in which the intervention should be realised.

**Fig. 3:**
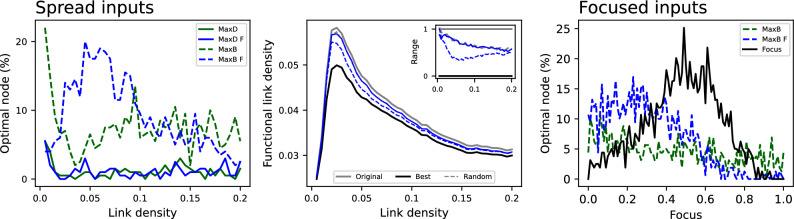
Interventions on random networks. (Left) Percentage of time the intervened node is the optimal one in terms of reducing the number of functional links, as a function of the link density in the physical layer. Results correspond to the targeting of the node with maximal degree (solid lines) or of maximal betweenness centrality (dashed lines), in the physical (green lines) and functional (blue lines) layers. (Centre) Evolution of the number of functional links, as a function of the link density. Lines correspond to the original dynamics (solid grey), the intervention on the optimal (solid black) and a random node (dashed grey), and the intervention on the node of maximal betweenness centrality in the physical (solid blue) and functional (dashed blue) layers. The inset represents the same results, normalised between the best and original (not intervened) cases. (Right) Percentage of time the intervened node is the optimal one as a function of the focus - see text for definition.

The left panel of Fig. 3 reports the evolution of the percentage of times in which a given strategy for selecting the node to be intervened yielded the best result, as a function of the link density of the physical network. As can be appreciated, selecting the node of maximal out-degree, both in the physical (green solid line) and in the functional (blue solid line) representations, is almost never the optimal solution. Better results can be obtained when targeting nodes with the highest betweenness centrality, mostly in the functional representation (dashed blue line), but also in the physical one (dashed green line). This is partly to be expected, as the betweenness is a topological centrality metric specifically designed to represent the importance of nodes in diffusion processes. At the same time, it is worth noting that this strategy is the best one only in the $20\%$ of cases at best.

These results are further confirmed in the central panel of Fig. 3, depicting the evolution of the link density in the functional layer as a function of the link density in the physical one. Note that the number of functional links is here used as a proxy of the intensity of the propagation process. Interventions on the node of highest betweenness centrality in the functional network can significantly reduce the propagation, midway between what obtained in optimal and random interventions, especially for low link densities.

The previous model assumed that the elements to be propagated could appear uniformly across all nodes; this is not always realistic, as some nodes may be more important, e.g. may provide more passengers or goods to the system. We account for this by introducing a focus factor, defined between zero and one, defining the probability for the elements to be generated in a specific (fixed) node. Hence, a focus of one implies that input elements always appear in the same focus node; on the other hand, a focus of zero recovers the previous uniform situation. As can be seen in the right panel of Fig. 3, targeting nodes with the highest betweenness centrality in the functional layer (dashed blue line) is still a good strategy. Still, substantially better results could be achieved if the focus node would be known and targeted, see solid black line.

### Modular physical networks

As a further step towards physical topologies more representative of real systems, we introduce a modular structure in the previously described random model^30,31^. Specifically, nodes are equally distributed in two communities; links initially connect nodes of the same community, according to a random topology and with a fixed link density of 0.05. We next add a number of bidirectional links between the two communities, connecting random nodes, hence strengthening the inter-community connectivity and progressively reducing the modularity. We finally measure the intensity of the propagation process through the number of reconstructed functional links; yet, as the interest is here in the modular structure of the network, we will specifically focus on the number of those links connecting the two communities.

**Fig. 4:**
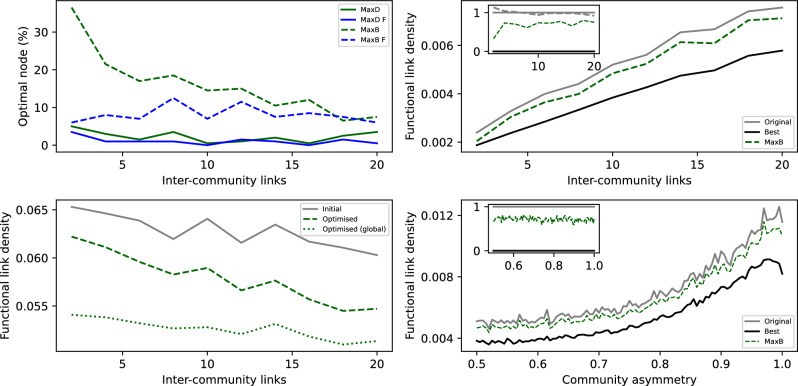
Interventions on physical layers with a modular structure. (Top left) Evolution of the percentage of time the intervened node is the optimal one, as a function of the number of inter-community links. Meaning of lines is the same as in the left panel of Fig. 3. (Top right) Evolution of the number of functional links as a function of the number of inter-community links. (Bottom left) Evolution of the number of functional links as a function of the number of inter-community links, when the intervention is targeted to reduce the number of propagations across communities. (Bottom right) Evolution of the number of functional links as a function of the community asymmetry, i.e. the probability that the elements to be propagated will appear in the same community.

The top left panel of Fig. 4 reports the evolution of the percentage of times that selecting a node of a given property is the best strategy for mitigating the propagation - that is, the same as in the left panel of Fig. 3, but here as a function of the number of inter-community links. Notably, while the betweenness of nodes is still the most important metric, better results are now obtained when this is calculated on the physical network (dashed green line). This is partly to be expected: especially for few inter-community links, nodes of high betweenness should be the one connecting the two communities. At the same time, this entails a challenge, as the physical network (i.e. the network that drives the propagation process) is assumed to be unknown and not observable.

Even if the physical network were known, intervening on the node with the highest betweenness centrality has a limited impact in the inter-community propagation, especially when the number of links connecting the two communities increases - see top right panel of Fig. 4. Notably, it has positive effects on the global propagation. To illustrate, in the bottom left panel of Fig. 4 we report the evolution of the total initial number of functional links (solid grey line), i.e. both intra- and inter-community ones; the total number of functional links, when intervening on the node that minimises the inter-community propagation (dashed green line); and the same metric, when intervening on the best node overall (dotted green line). It can be appreciated that targeting the inter-community propagation yields results not far from what obtained when targeting the global propagation, especially for high inter-community connectivities.

As a final point, we evaluate what happens if the elements to be propagated tend to appear mostly in one community, i.e. in an asymmetric way. The bottom right panel of Fig. 4 reports the evolution of several metrics as a function of this asymmetry, i.e. of the probability with which elements appear in one of the two communities. In a way that may be unexpected, the higher such asymmetry, the larger is the number of inter-community functional links. This is nevertheless easily explained: when elements can only appear in one community, the fact that we observe them in the other community must necessarily be the result of a inter-community propagation; hence, these latter links are easy to be detected, as they are not masked by the sudden appearance of elements in the second community. On the other hand, the asymmetry has no impact on the fraction of propagation that we can avoid by intervening on the node with highest betweenness centrality in the physical network - see inset.

### Scale-free physical networks

As a last synthetic model, we consider a physical network with a scale-free structure, i.e. whose cumulative degree distribution of nodes follows a power law: $P(k) = c \cdot k^{-\lambda }$. As is the case of modularity, scale-freeness is a property commonly found in many real-world networks, both natural and manmade^32–34^. These networks are here generated by defining a sequence of node degrees, according to a power law of exponent $\lambda$; for then creating the physical network using a configuration model on that sequence^35,36^. As in previous cases, the number of nodes is set to $N = 100$.

**Fig. 5:**
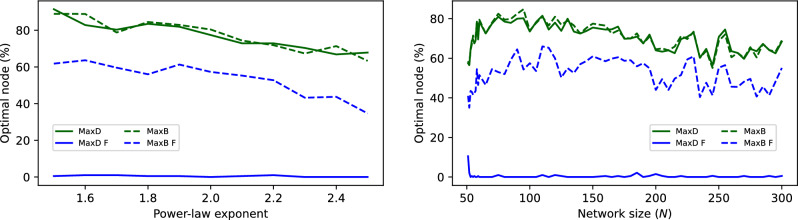
Interventions on physical layers with a scale-free structure. (Left) Evolution of the percentage of time the intervened node is the optimal one, as a function of the exponent of the degree distribution. Meaning of lines is the same as in the left panel of Fig. 3. (Right) Evolution of the same metrics, for $\gamma = 2$, as a function of the number of nodes *N*.

Results are reported in Fig. 5 following the usual colour schema, as a function of the power law exponent $\lambda$. When analysing the percentage of time a node with a given property is the optimal one for an intervention (see left panel), results are quite clear: properties of the physical network (green lines) are much more important than those of the functional one (blue lines); and the degree of nodes, while being important in the former case (solid green line), is completely irrelevant in the latter (solid blue line). In other words, the optimal solution to reduce the propagation is to act on the node of highest degree in the physical network, i.e. on its hub. Notably, this is highly consistent, being this the optimal node on average $80\%$ of the time; but, at the same time, the physical topology, and hence the identity of this node, is not known. On the contrary, intervening on the node of highest degree in the functional network, i.e. the strategy that may intuitively be followed, is never the optimal solution. Note that the limited size of the scale-free networks here considered may be introducing some biases, as this property usually requires an evaluation of the degree over multiple orders of magnitude. Results are nevertheless stable even for higher numbers of nodes *N*, see right panel of Fig. 5.

### Analysis of the European air transport network

As a final case study, we evaluate how the previously described ideas apply in the case of the European air transport network, and specifically the propagation of delays therein. This is one of the most important topics in air transport research, as delays have major negative impacts on the cost-efficiency^37^, safety^38^, and environmental footprint of aviation^39^; and are responsible for major societal costs, not only monetary but also in terms of negative public opinion^40^.

**Fig. 6:**
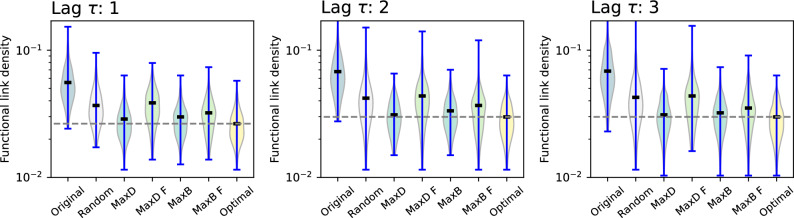
Analysis of the propagation of delays in the European air transport network, with random delays. From left to right, the three panels report the distribution of the density of functional links, as a function of the intervention strategy, for three values of the lag $\tau$. The horizontal dashed lines report the best achievable result, for visual reference.

A distinction is usually made between primary and secondary delays: the former ones are the result of random and unpredictable internal processes or external events, as e.g. adverse weather; the latter ones emerge when primary delays cause further delays for other aircraft and flights^41^. Our interest is here focused on secondary ones, also known as *reactionary* or *knock-on* delays, as they are the result of a propagation. Note that the true propagation of delays (i.e. the underlying network) is not known; modelling such phenomenon^42–44^, and the reconstruction of the network created by such propagation^10,27,45,46^, are active topics of research. Therefore, in what follows we will suppose that delays propagate following the same structure as flights; two airports strongly connected by direct flights will have a higher probability of also sharing delays. The starting point is the network created by the top-30 European airports, in terms of number of operations; and their connectivity is given by the number of direct flights between them, as of September 2019 - note that this corresponds to the month with the largest number of operations before the appearance of COVID-19 in the following year. All data have been obtained from the EUROCONTROL’s R&D Data Archive, a public repository of historical flights made available for research purposes and freely accessible at https://www.eurocontrol.int/dashboard/rnd-data-archive.

We start by considering a basic scenario, in which delays are generated in a random way and uniformly across airports - i.e. all airports have the same probability of experiencing a new delay, thus being equivalent to the model of Fig. 2. The model is then executed 200 times, each time using the connectivity network of a random day of September 2019 - this is included to account for the natural variability of flight scheduling across week days. Next, following the previous analyses, the corresponding functional networks are reconstructed. As the last step, the resulting functional link density is evaluated and depicted in Fig. 6, both in the original configuration, and when different mitigation strategies are deployed.

Some interesting insights can be extracted from Fig. 6. First of all, as may be expected, manipulations can substantially reduce the amount of functional links and hence of propagated delays. This is especially true in the optimal case, i.e. when the node chosen for the intervention is the one that will mostly impact the propagation, with a two-fold reduction. Note that this is not trivial nor given: due to the high interconnectivity of the network and its complex topology, the fact that such a positive impact can be achieved by only intervening on one airport is noteworthy. At the same time, good results can be obtained by following topological metrics of the physical layer, but less good when using the functional network. This is something negative, as the real topology of propagation is not known, and policymakers may only rely on the reconstructed functional layer.

**Fig. 7:**
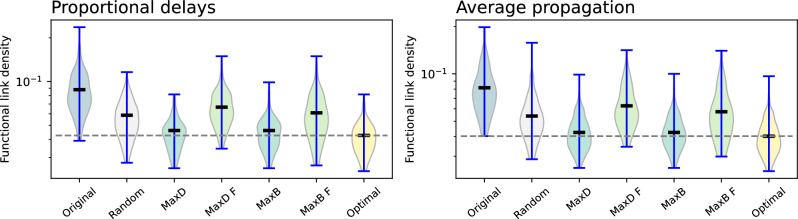
Analysis of the propagation of delays in the European air transport network, with delays appearing in a way proportional to the size of each airport. The right panel corresponds to the same model, but using the total propagation network for September 2019. Each panel reports the distribution of the density of functional links as a function of the intervention strategy, for $\tau = 1$. The horizontal dashed lines report the best achievable result, for visual reference.

We further evaluate the dynamics of the system in two additional conditions. Firstly, we change the probability of a delay appearing at one airport to be proportional to the number of flights there operated. This is more realistic, as large airports are usually saturated, i.e. working at the limit of their capacity; any disruption there appearing can have a large impact. Secondly, we consider the same scenario, but in every realisation of the model we consider a propagation network equal to the average across all days of September 2019. Results are respectively represented in the left and right panels of Fig. 7. Compared to the previous results, one important difference stands out: while the use of topological metrics of the propagation (physical) network yields near-optimal results, the opposite happens when considering the functional layer. This behaviour is easy to explain: as delays mostly appear in large airports, intervening these is a good strategy. Yet, nodes with high degree in the functional layer may not be the same as those in the physical layer, as shown in Fig. 2; hence, intervening on the former is not the optimal solution. These results will be used below to draw some operational conclusions.

**Fig. 8:**
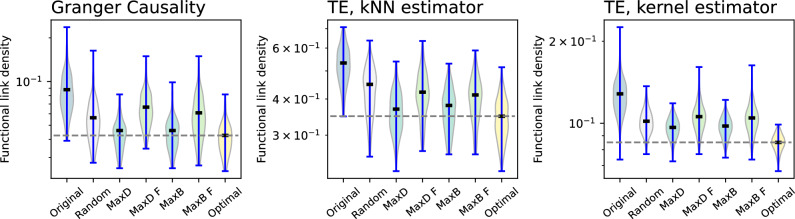
Propagation of delays according to different causality metrics. Each panel reports the distribution of the density of functional links as a function of the intervention strategy, for $\tau = 1$. From left to right, results correspond to the use of Granger Causality, thus being identical to the left panel of Fig. 7; Transfer Entropy with a kNN estimator; and Transfer Entropy with a kernel estimator.

As a final topic, it is worth noting that these results have been obtained through the celebrated Granger Causality; while this was chosen for its conceptual simplicity and prevalence, results might not be generalisable to different causality metrics. In order to check this, Fig. 8 reports the results obtained from the model of delay propagations in the European air transport network with $\tau = 1$, changing the way functional links are reconstructed. Specifically, three cases are considered: the previous Granger Causality (left panel, hence being identical to the left panel of Fig. 7), Transfer Entropy^47^ with a kNN estimator of the underlying data distributions^48^ (central panel), and Transfer Entropy with a kernel estimator^49^ (right panel). In both cases, the implementation of the Transfer Entropy corresponds to the *InfoMeasure* library, see Ref.^50^. While results in the three panels of Fig. 8 have different magnitudes, i.e. each causality metric detects a different share of links as relevant, the global trend is preserved. In other words, topological features are not reliable guides to intervene on the system also in the case of functional networks reconstructed using Transfer Entropy.

## Discussion and conclusions

Can functional networks be trusted to correctly guide the intervention on a complex system? In this contribution we tried to answer this question by resorting to a minimal model, comprising a physical (unknown to the researcher) layer on top of which a propagation process takes place; the reconstruction of the corresponding functional representation; and the evaluation of interventions based on node properties. In general, the answer to this question seems to be negative. The results here obtained indicate that intervening on the nodes most central in the functional network works well in the case of random networks (see Fig. 3); but conversely, yields sub-optimal results in more complex topologies, e.g. modular (Fig. 4), scale-free (Fig. 5), or real ones (Figs. 6 and 7). In other words, when the physical propagation layer is uninformative, the functional connectivity patterns can help guiding the intervention and improve the process. Nevertheless, when the physical layer has some salient characteristics, it is always better to use these. This further seems to be independent on the specific causality metric (Fig. 8); and on other parameters, as e.g. the network size (right panel of Fig. 5). An explanation for these results may be the incapacity of the functional representation to identify nodes of high in-degree, as seen in Fig. 2, something more relevant in the case of heterogeneous networks. This is problematic, as we assume that the physical propagation layer is not know - as, otherwise, there would be no need to reconstruct the functional representation in the first place.

From the point of view of the use case here considered, this is especially problematic. As is to be expected for any real system, functional networks of delay propagations in air transport are not perfect; due to the limited availability of data and the amount of noise in the same, reconstructed functional networks may not perfectly reflect the real propagation patterns^27^, beyond the limitation previously discussed in e.g. Fig. 2. Air transport is further a highly dynamical and non-stationary system, adapting to seasonal and long-term changes in mobility demand; functional networks may therefore substantially change across time, limiting our ability of reconstructing them using long time series. On top of that, the complex topology of the network implies that using properties of the functional layer to guide interventions is not necessarily better than intervening airports at random (see Figs. 6, 7 and 8).

It may be tempting to conclude that functional networks are worthless in this context; truth is nevertheless more complex. The model here presented is a minimal one, intentionally designed to represent a general propagation process. To illustrate, delay propagation in air transport is a more complex phenomenon, driven by many factors beyond the number of flights, and which affect how two airports are connected - for instance, aircraft, crew and passenger connections, use of common resources (e.g. airspaces), and so forth. The fact that the physical propagation layer here considered does not correspond to reality results in differences in the functional layers. To illustrate, when a functional network is reconstructed using real delay data (for the same airports and time window, using GC and standard time series detrending procedures^10^), *p*-values obtained from the model and from the real data are only weakly correlated (linear Pearson’s correlation of $\rho = 0.229$). Even though the reconstructed functional networks are different, the general message is still valid: topological metrics of functional representations may be unreliable guides.

Another limitation of this negative conclusion may be connected to the way functional networks are evaluated. Even though the topological metrics here considered (degree and betweenness centrality) are prima facie relevant and represent the standard approach in the literature, better results may be obtained by designing new ones specifically tailored at this problem. In other words: the concept of centrality in functional networks may not coincide with what centrality is usually thought of in physical networks. These new topological metrics may further incorporate the idiosyncrasies of the propagation process under study.

## Data Availability

The dataset analysed during the current study is available in the EUROCONTROL’s R&D Data Archive repository, https://www.eurocontrol.int/dashboard/rnd-data-archive.
